# Short-term wind speed forecasting based on a hybrid model of ICEEMDAN, MFE, LSTM and informer

**DOI:** 10.1371/journal.pone.0289161

**Published:** 2023-09-08

**Authors:** Wang Xinxin, Shen Xiaopan, Ai Xueyi, Li Shijia

**Affiliations:** School of Management, Wuhan University of Science and Technoloy, Wuhan, China; Huazhong University of Science and Technology, CHINA

## Abstract

Wind energy, as a kind of environmentally friendly renewable energy, has attracted a lot of attention in recent decades. However, the security and stability of the power system is potentially affected by large-scale wind power grid due to the randomness and intermittence of wind speed. Therefore, accurate wind speed prediction is conductive to power system operation. A hybrid wind speed prediction model based on Improved Complete Ensemble Empirical Mode Decomposition with Adaptive Noise (ICEEMDAN), Multiscale Fuzzy Entropy (MFE), Long short-term memory (LSTM) and INFORMER is proposed in this paper. Firstly, the wind speed data are decomposed into multiple intrinsic mode functions (IMFs) by ICEEMDAN. Then, the MFE values of each mode are calculated, and the modes with similar MFE values are aggregated to obtain new subsequences. Finally, each subsequence is predicted by informer and LSTM, each sequence selects the one with better performance than the two predictors, and the prediction results of each subsequence are superimposed to obtain the final prediction results. The proposed hybrid model is also compared with other seven related models based on four evaluation metrics under different prediction periods to verify its validity and applicability. The experimental results indicate that the proposed hybrid model based on ICEEMDAN, MFE, LSTM and INFORMER exhibits higher accuracy and greater applicability.

## Introduction

With the rapid growth of the world economy, environmental and resource scarcity is becoming increasingly serious. Currently, the world is suffering from resource scarcity and ecological shock as well as energy and environmental challenges, making it quite urgent to transform traditional energy structure and reduce dependence on fossil fuels [1]. Wind energy, a kind of green and sustainable energy, is very environmentally friendly and does not generate any harmful substance compared with traditional fossil fuels. Plus, wind power is becoming more and more cost-effective, making it more and more important for improving energy structure and addressing environmental pollution. Wind speed prediction holds crucial practical significance in the efficient management and optimization of wind power systems. An increasing number of countries have begun their attempts to develop wind energy, among which China is the world’s largest market and produce for wind energy. By the end of 2021, China’s installed wind power capacity has reached 290 GW, accounting for 42.3% of the global total installed capacity [2].

However, unstable and intermittent wind speed causes fluctuations in the generation of wind power and affects the utilization of wind energy, seriously hindering the advance of wind power generation. Therefore, accurate wind speed prediction is crucial to improving the efficiency and stability of wind power generation [3]. The change in the wind speed needs to be predicted so that wind turbine can be regulated prior to such change to maximize the utilization of wind energy while reducing the fluctuation and instability caused by changing wind speed.

Currently, wind speed is mainly predicted by physical model, statistical model, machine learning model, and hybrid model.

Physical model approach refers to Numerical Weather Prediction (NWP). NWP technology is a mathematical and physical model-based weather forecasting technique that simulates atmospheric motion through mathematical models to predict weather changes. Based on physics, this technology divides the atmosphere into countless grid points, and then simulates atmospheric motion through computers and solves weather equations using numerical methods to derive weather changes in the future time period. Zhao et al. presented a day-ahead probabilistic wind speed prediction model based on the optimized NWP to achieve probabilistic one-day 96-step wind speed prediction [4]. Based on a single NWP wind speed prediction model, He et al. proposed a short-term wind power prediction model that combines deep learning models with numerical weather prediction for wind speed prediction. The model was designed to predict wind power accurately under different weather conditions [5]. However, the NWP model is complex and the data is difficult to determine, which makes it challenging to control errors in different stages. Consequently, prediction results are prone to bias [6].

Statistical model is a method of predicting future wind speed based on historical data. This model predicts future wind speed using historical data based on time series analysis or regression analysis. The models based on time series analysis include Bayesian model [7], autoregressive integrated moving average model (ARIMA) [8], auto regression moving average (ARMA) [9], and generalized autoregressive conditional heteroskedasticity model (GARCH) [10], while those based on regression analysis include linear regression, logistic regression and multiple regression models. Aasim et al. proposed a new RWT-ARIMA model, which was validated to have good performance in the short-term prediction of wind speed [11]. García et al. put forward a one-by-one truncated binary matrix Bayesian dynamic linear model for joint wind component analysis and short-term wind prediction and verified the prediction performance of their model [12]. Jiang et al. established a hybrid GARCH-based prediction method to facilitate wind speed prediction, which can better capture the fluctuation in self-sequences [10]. The statistical model is easy to interrupt, susceptible to outliers and trends and difficult to capture nonlinear signals despite the fact that it is simple and easy to use.

In recent years, machine learning and deep learning methods have been widely used in wind speed prediction, including Recurrent Neural Network (RNN) [13], Long Short-term memory (LSTM) [14], Convolutional Neural Network (CNN) [15], support vector machine (SVM) [16], and Transformer [17]. These artificial intelligence-based methods can better handle complex nonlinear relationships and multimodal data in wind speed prediction, thus improving prediction accuracy and efficiency. Among these machine learning and deep learning algorithms, LSTM performs well as an improved RNN algorithm, which has good performance in short-term wind power prediction. Banik et al. proposed a deep learning algorithm based on LSTM to predict short-term wind speeds [18]. Memarzadeh et al. proposed a new hybrid forecasting model for short-term power load and price forecasting based on LSTM [19]. LSTM has demonstrated strong performance in short-term prediction tasks attributed to its unique gating mechanism. However, its efficacy diminishes when applied to long-term prediction scenarios. Studies have shown that the prediction speed of LSTM decreases rapidly and that of MSE increases rapidly after predicting more than 48 points. In the case of an short prediction interval (minutes or seconds), only 48 periods of data may be far from sufficient, and LSTM is not capable to parallel learning [20]. Transformer has also received a lot of attention since its introduction, and is suitable for long-order prediction and can learn in parallel [21, 22]. However, Transformer suffers from complicated calculation, high memory consumption and slow speed. Informer proposes the ProbSparse self-attention mechanism on the basis of Transformer to overcome temporal and spatial complexity while overcoming the deficiencies of computational complexity and slow speed, and is applicable to the prediction of wind speed at different time periods [20]. Bai et al. employed the Informer model for medium to long-term wind power prediction and substantiated its superiority in terms of reduced prediction errors and enhanced performance for long time series power prediction tasks [23]. Huang et al. used Multi-step informer for medium to long-term wind power forecasting. The performance of Informer in medium to long-term prediction is notable [24]. The above research indicates that methods based on deep learning performs well in predicting wind speed, but those based on single deep learning is far from sufficient to handle non-stationary and fluctuating wind speed data to meet the accuracy requirement. And with the explosive growth of data volume, it is difficult to explore the intrinsic deep features of wind speed by single model, making it extremely important to preprocess data.

To further improve the accuracy of wind speed prediction, more and more hybrid prediction models have started to show their advantages in recent years. Hybrid prediction models are mainly divided into three categories. Firstly, the prediction accuracy of a single model can be somewhat improved by combining different predictors [13, 25]. Combining CNN and Bi-LSTM, Nguyen et al. not only extracted the internal features of time series, but also fully exploited forward and backward information [26]. Wang et al. developed a multivariate combined wind speed prediction system based on convolutional and recurrent neural networks [27]. The second type of hybrid model was composed of optimization algorithm and predictor, which could effectively improve the prediction performance of the model by optimizing the parameters of the predictor through the optimization algorithm [28–32]. Wang et al. combined extreme learning machine (ELM) with AdaBoost algorithm and used automatic weather station data to select different locations as target stations for multi-timescale wind speed prediction [33]. ElKenawy et al. proposed a high-precision wind speed prediction method, and optimized the hyperparameter of various models with ADGWDTO algorithm [28]. Xian et al. proposed a multi-kernel SVR ensemble (MKSVRE) model based on unified optimization and whale optimization algorithm (WOA) and verified its effectiveness [34]. Finally, owing to complex and unstable wind series, data preprocessing methods, represented by data decomposition, have attracted intensive attention in recent years [35–37]. Wu et al. used the ensemble empirical modal decomposition (EEMD) to convert the 1-dimensional series of raw wind speed into 16-dimensional series, and directly modeled multidimensional wind speed data with Transformer [38]. Bommidi et al. Employing an improved complete ensemble empirical modal decomposition of adaptive noise (ICEEMDAN) decomposition method to denoise wind speed data [17]. However, the decomposition of wind speed sequences introduces multiple modes, thereby increasing computational complexity. Consequently, scholars have employed calculations of sample entropy (SE) and fuzzy entropy (FE) to assess the complexity of the time series. It has been observed that higher entropy values indicate a higher level of complexity in the time series. To mitigate the computational burden, researchers have effectively reduced the complexity by merging sequences with similar entropy values, while ensuring the preservation of prediction accuracy [39, 40]. Qin et al. reorganized the components by fuzzy entropy [41]. Peng et al. performed decomposition and reconstruction by combining OVMD with SampEn [42]. In addition, scholars have considered wind speed prediction models with multivariate and multi-objective optimization. Lv et al used improved hybrid time series decomposition strategy (HTD), novel multi-objective binary backtracking search algorithm (MOBBSA) and advanced Sequence-to-Sequence (Seq2Seq) predictor to extract, decompose, and predict features of wind speed sequences, and verified its effectiveness [43]. Considering the influence of various meteorological factors, Lv et al. designed a filter-wrapper non-dominated sorting differential evolution integrating K-medoid clustering (FWNSDEC) to generate feature subsets. Then, effective prediction of the three-dimensional sequence sample set is achieved through singular spectrum analysis and convolutional long short-term memory (ConvLSTM) networks [44]. Some representative literatures can be seen in Table 1.

**Table 1:** Some representative literatures on the prediction model.

Prediction Model Category	Reference	Strengths	Weaknesses
Physical model	NWP [4, 5]	Wide spatial range; long time range	Limited accuracy; complexity
Statistical model	ARMA [9]; ARIMA [8] GARCH [10]; RWT-ARIMA [11]	Relatively simple, easy to use	Cannot capture the nonlinear characteristics
Single machine learning/ deep learning	SVM [16]; CNN [15]; RNN [13]; LSTM [14]	Able to capture nonlinear signals	High demand for data, easily to fall into local optimum
Fusion model based on deep learning and optimization algorithms	FWA-LSTM [32]; CNN-LSTM [26]; ELM-AdaBoost [33]; WOA-SVR [34]	Optimize predictor parameters, enhanced predictive performance	High computing costs, not suitable for long-term times series
Fusion model based on deep learning and decomposition	EMD [37]; VMD [2]; EEMD [38]; ICEEMDAN [17]	High complexity, can effectively capture nonlinear signals	High computing costs, not suitable for long-term times series
Transformer	[17, 21, 31]	Suitable for long-term times series	Complex model, hard to train, high demand for data
Informer	[20, 23, 24]	Suitable for long-term times series, reduced computational complexity on the basis of transformer	High demand for data

According to the research, hybrid models are a viable solution to overcome the limitations of single models while achieving high levels of accuracy. Specifically, the hybrid framework that incorporates data preprocessing and multiple predictors has been found to perform well in this respect. This approach provides a comprehensive and nuanced perspective, emphasizing the unique strengths of each component to enhance prediction accuracy and practicality.

In decomposition, ICEEMDAN can better solve the problem of modal aliasing compared to other methods [17]; Multi scale fuzzy entropy can better measure the complexity of time series at different scales by utilizing multi scale coarse granularity [40]; INFORMER and LSTM perform well in long-term times series and short-term times series, respectively [14, 23].

However, most of the existing research on wind speed prediction is only suitable for short-term wind speed prediction. As the number of prediction points increases, its prediction accuracy decreases rapidly. Therefore, based on the above considerations, this paper proposes a new hybrid prediction model that combines ICEEMDAN, MFE, LSTM and INFORMER to improve the prediction accuracy and applicability.

The contributions of this paper are mainly as follows.

1. ICEEMDAN is used to decompose the original wind speed series into multiple IMF components to reduce the prediction difficulty and denoising.
2. Under the premise of ensuring the prediction performance, the MFE values of each IMF component are calculated to reduce the computational effort, and the components with similar MFE are combined to generate new subseries.
3. The INFORMER and LSTM model is used to predict each subseries, and then the results are superimposed to obtain the final predicted wind speed.
4. The effectiveness and predictive performance of the proposed model were evaluated by comparing it to seven other related prediction models. Additionally, to validate the long sequence advantage of the INFORMER prediction model, a comparison was made with the LSTM model, and the predictive performance of both models was analyzed under different prediction points.

The rest of this paper is arranged as follows: Section 2 explains each method involved in the proposed hybrid model, including ICEEMDAN, MFE, LSTM and INFORMER. Section 3 describes the overall architecture of the hybrid model. Section 4 analyzes and validates the effectiveness of the proposed model through a real case. Section 5 concludes the whole paper.

## Methodology

This section introduces the principles and excellence of various algorithms used in the prediction model. The selected model includes improved fully adaptive noise ensemble empirical modal decomposition, multiscale fuzzy entropy, and informer.

### Improved Complete EEMD with Adaptive Noise (ICEEMDAN)

#### Empirical Mode Decomposition

Empirical Mode Decomposition (EMD) is a signal processing technique used to decompose a signal into a set of fixed-shaped eigenmode functions known as Intrinsic Mode Functions (IMFs). The main concept behind EMD is to decompose the original signal into several IMFs, each of which is locally smoothed on the time scale and has frequency components that vary with the scale.

The EMD algorithm involves several steps. Firstly, the envelope is extracted from the original signal to obtain an envelope curve. Secondly, the envelope curve is subtracted from the original signal to obtain a residual signal. Finally, the residual signal is summed up to obtain an approximation of the original signal.

Despite the advantages of EMD, it has several drawbacks. Firstly, the EMD algorithm requires several iterations and has a large computational effort, which makes the algorithm slow and difficult to handle large-scale data. Secondly, the eigenmodes are extracted by solving the envelope and local average, but at the local extremes, both the envelope and the local average may fail, leading to errors in the extraction of the eigenmodes. Lastly, the EMD algorithm is sensitive to noise because noise can interfere with the local features of the signal, leading to errors in the extraction of the eigenmodes.

In summary, EMD is a powerful signal processing technique that can decompose a signal into IMFs. However, it has several limitations, including computational effort, sensitivity to noise, and the local extremum problem, which can impact its effectiveness for some applications.

#### EMD related improvements

Several improved algorithms have been proposed to address the limitations and shortcomings of the Empirical Mode Decomposition (EMD) algorithm. For instance, the Ensemble Empirical Mode Decomposition (EEMD) and Complete Ensemble Empirical Mode Decomposition (CEEMD) algorithms add pairs of positive and negative Gaussian white noise to the signal to be decomposed, which mitigates the modal mixing problem of the EMD algorithm. The CEEMDAN (Complete Ensemble Empirical Mode Decomposition with Adaptive Noise) algorithm further improves on the EEMD algorithm by introducing the concept of adaptive noise, which makes the algorithm more robust to noise.

Despite the improvements made by these algorithms, they still have some limitations. The EEMD and CEEMD decomposition algorithms introduce a certain amount of white noise into the eigenmodal components, which can affect the subsequent analysis and processing of the signal. Additionally, the CEEMDAN algorithm may produce Intrinsic Mode Functions (IMFs) containing more noise, and discarding them directly after decomposition can result in the loss of useful information.

In summary, these improved algorithms have enhanced the reliability and accuracy of the EMD algorithm in different aspects. However, they still have some challenges that require further research and solution. For instance, efforts should be made to reduce the residual noise and pseudo-modal problems.

#### Improved complete EEMD with adaptive noise

Improved Complete EEMD with Adaptive Noise (ICEEMDAN) is an improved algorithm in Complete EEMD with Adaptive Noise (CEEMDAN) [39]. Different from CEEMDAN, Gaussian white noise is directly added in the decomposition process, but the kth IMF component is selected after the white noise is decomposed by EMD, mainly to solve the residual noise and pseudo-modal problems in CEEMDAN.

**Fig 1:**
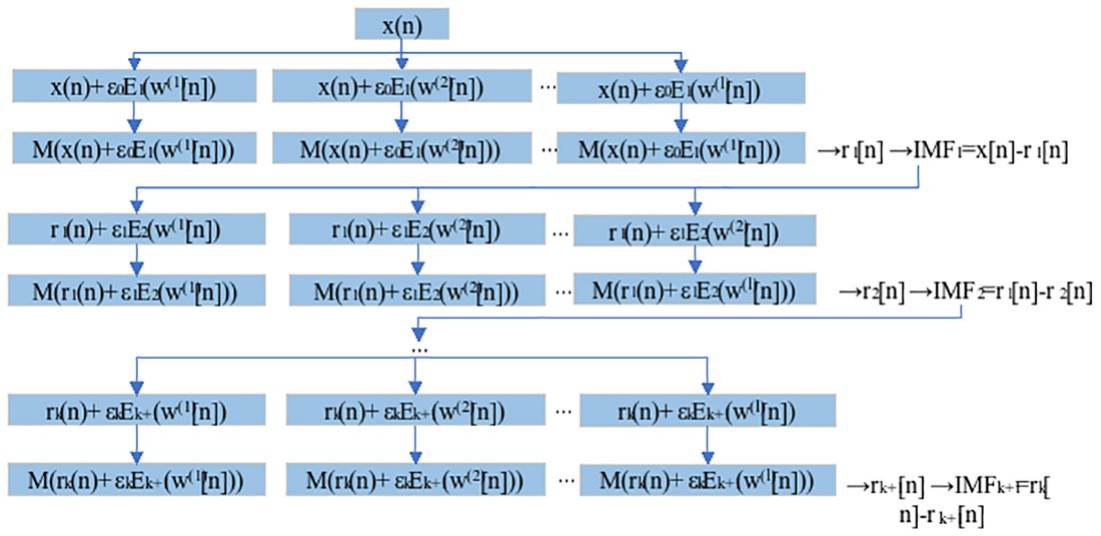
Calculation process of ICEEMDAN.

In ICEEMDAN, the operator *E*_*K*_(·) is the *k*_th_ order modal component after decomposition by EMD, and the operator *M*(·) is the local mean of the signal, which proceeds as shown in Fig 1.

As shown in the figure, *ω*^*(i)*^*[n]* is the ith group of Gaussian white noise added, so in each round of the IMF solving process, the noise added is the IMF component of the original noise signal. *ε*_*j*_ is the coefficient multiplied after the addition of noise component, and *E*_*k (*_*ω*^*(i)*^*[n])* is the kth EMD component of *ω*^*(i)*^*[n]*. The specific steps are as follows.

(1) After the addition of white noise to the original time series *x(n)*, the signal is *x*^*(l)*^*(n)*.
$$x^{\left(l\right)}(n)=x(n)+\varepsilon_{0}E_{1}(w^{\left(1\right)}[n])$$ (1)
(2) *x*^*(l)*^*(n)* is obtained by EMD decomposition of the 1_st_ residual component and the IMF component.
$$r_{1}(n)=M(x^{\left(l\right)}(n))$$ (2)
$$IMF_{1}(n)=x(n)-r_{1}(n)$$ (3)
(3) By analogy, the (*k+1*)_st_ residual component and the k_th_ IMF component can be expressed as:
$$r_{k}{}_{+1}(n)=M(r_{k}(n)+\varepsilon_{k}E_{k+1}(w^{\left(l\right)}(n)))$$ (4)
$$IMF_{k}(n)=r_{k}(n)-r_{k}{}_{+1}(n)$$ (5)
(4) Repeat step 3 until the residual component *r*_*k*_*(n)* is a monotonic function, then the original signal *x(n)* can be expressed as:
$$x(n)=\sum_{k=1}^{N}IMF_{k}(n)+r_{N}(n)$$ (6)
  where N is the number of decomposed components.

By incorporating an adaptive noise mechanism, ICEEMDAN dynamically adjusts the noise level based on signal characteristics and noise intensity. This adaptive feature effectively mitigates noise interference during signal decomposition, thereby enhancing the accuracy and robustness of the process. Additionally, ICEEMDAN decomposes the signal into multiple intrinsic mode components using EEMD, with each component maintaining independence. This independence allows each IMF to accurately represent different signal components, thus preventing aliasing and interference among them. In summary, the combination of these characteristics establishes ICEEMDAN as an effective and reliable method for signal decomposition.

### Multiscale fuzzy entropy

Multiscale fuzzy entropy (MFE) is a method that combines fuzzy entropy and multiscale entropy to better measure the complexity of time series at different scales. While basic fuzzy entropy has limitations due to the fixed sampling rate and single scale, MFE introduces a multiscale coarse-graining process to provide an additional observation perspective when the time scale is uncertain. By utilizing multiscale coarse-graining, MFE captures more information about the signal, allowing for a more accurate measurement of complexity.

#### Fuzzy entropy

Fuzzy Entropy and sample entropy are similar in the sense that both measure the probability for a time series to generate a new pattern in the case of the change in dimensionality. The higher the probability for the series to generate a new pattern, the more complex it will be, and the higher the entropy value. The calculation steps are as follows.

Suppose there is a time series consisting of N data, *x(n) = x(1)*, *x(2)*, *x(3)*, *…x(n)*, then the FE value is denoted as *FE(m*,*r*,*n)*, where *m* is the Embedding Dimension and *r* is the similarity tolerance threshold which generally ranges from 0.1to 0.25std(x). The FE calculation steps are specifically described as follows.

(1) Sequential segmentation.
  Form a sequence of vectors of dimension *m* by serial number.
$$X_{i}{}^{\text{m}}=\{X_{1}{}^{m},X_{i+1}{}^{m},\ldots \ldots ,X_{N-m+1}{}^{m}\},(i=1,2,3\ldots \ldots ,N-m+1)$$ (7)
$$X_{i}{}^{\text{m}}=\{x(i),x(i+1),\ldots \ldots ,x(i+m-1)\}-x_{m}{}^{i},(i=1,2,3\ldots \ldots ,N-m+1)$$ (8)
  where *x*_*m*_^*i*^ is the mean value of *X*_*i*_^*m*^.
$$x_{i}{}^{m}=\frac{1}{m}\sum_{k=0}^{m-1}x(i+k)$$ (9)
(2) Define the distance *d*_*ij*_^*m*^ between the vector *X*_*i*_^*m*^ and *X*_*j*_^*m*^ as the Chebyshev distance, i.e., the maximum of the absolute value of the difference between the values of the elements.
$$d_{ij}{}^{m}=D_{chebychev}(X_{i}{}^{m},X_{j}{}^{m})={\text{max}}_{k=0,1,2,\ldots \ldots ,m-1}\left|x(i+k)-x(j+k)\right|$$ (10)
(3) Define the similarity: Introduce the fuzzy affiliation degree *n* and measure the similarity between *X*_*i*_^*m*^ and *X*_*j*_^*m*^.
$$D_{ij}{}^{m,n,r}=e^{-{\frac{\left(d_{i}{{}_{j}}^{m}\right)}{r}}^{n}},1\leq j\leq N-m,j\neq i$$ (11)
(4) Define the function *O*^*m*,*r*,*n*^.
$$O^{m,r,n}=\frac{1}{N-m}\sum_{i=1}^{N-m}\left[\frac{1}{N-m-1}\sum_{j=1,j\neq i}^{N-m}D_{ij}{}^{m,n,r}\right]$$ (12)
(5) Reconstruct a set of vectors of dimension m+1 and calculate the similarity.
$$O^{m+1,r,n}=\frac{1}{N-m}\sum_{i=1}^{N-m}\left[\frac{1}{N-m-1}\sum_{j=1,j\neq i}^{N-m}D_{ij}{}^{m+1,n,r}\right]$$ (13)
(6) Define the fuzzy entropy: The fuzzy entropy *FE(m*,*r*,*n)* of the sequence is expressed as:
$$FE(m,r,n)=\text{ln}\;O^{m,r,n}-\text{ln}\;O^{m+1,r,n}$$ (14)

The fuzzy entropy of time series can be calculated according to the above steps.

#### Multiscale fuzzy entropy

Multiscale fuzzy entropy draws on the idea of multiscale entropy [40]. ME is designed to measure the complexity and self-similarity of time series at different scale factors. If the entropy value of one sequence is higher than that of the other at most scale entropy values, the former is more complex than the latter. If the entropy value of a time series decreases monotonically with the increase in scale factor, then the structure of the series is relatively simple. The MFE calculation procedure is as follows.

(1) For a discrete time series *X*_*i*_ of length N, given the embedding dimension *m* and the similarity tolerance *r*, a new coarse grained vector *y*_*k*_^*τ*^ is constructed based on the original sequence.
$$y_{k,j}^{\tau }=\frac{1}{\tau }\sum_{i=(j-1)\tau +k}^{j\tau +k-1}X(i),1\leq j\leq \frac{N}{\tau },1\leq k\leq \tau$$ (15)
  where *τ* is the scale factor. When *τ* = 1, the coarse-grained time series is the original sequence *X*_*i*_, and when *τ* ≠ 0, the original sequence *X*_*i*_ is decomposed into coarse-grained sequences *y*_*k*_^*τ*^, *τ* is the number of decomposition, and N/*τ* is the length of each segment.
(2) The fuzzy entropy FE is calculated according to *τ* and is listed as a function of the scale factor with constant similarity tolerance *r*, which is usually taken as 0.1–0.25 times the standard deviation of the original sequence.

Based on the above steps, the MFE can be expressed as:

$$MFE(X,\tau ,m,r)=FE(m,r,y_{k}^{\tau })$$ (16)

#### Informer network

**Fig 2:**
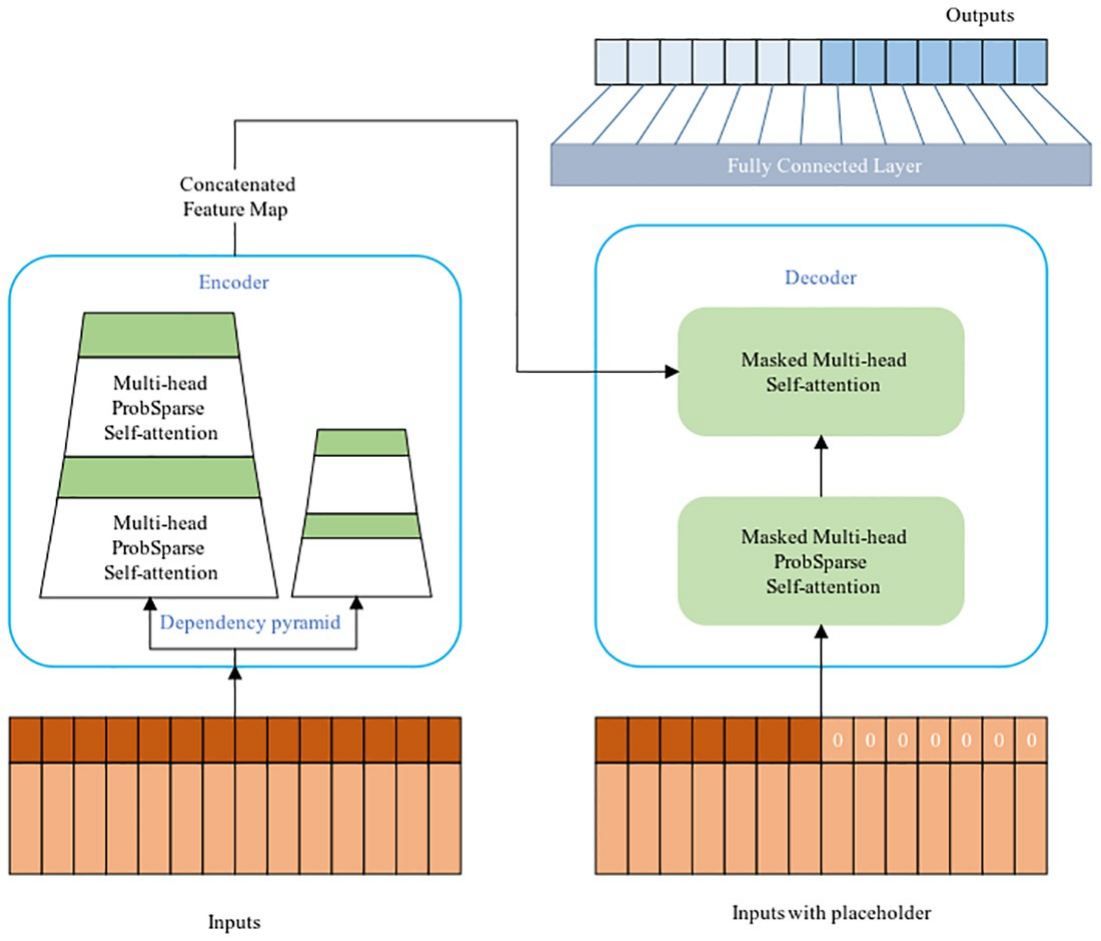
Informer model.

The informer is composed of an encoder and a decoder. The encoder handles long input sequences and reduce time complexity through probspare self-attention. By incorporating distillation in self-attention, the encoder effectively reduces the time dimension of input sequences. In addition, the generative decoder can generate the final results in one step instead of one step at a time. The overall structure is shown in Fig 2 [20].

*(1) ProbSpare self-attention*. The traditional self-attentive mechanism involves three inputs, namely query, key and value, and the attention matrix of the inputs is calculated using deflated points.

$$Atten(Q,K,V)=Softmax(\frac{QK^{T}}{\sqrt{d}})V,Q\in R^{L_{Q}\times d},K\in R^{L_{K}\times d},V\in R^{L_{V}\times d}$$ (17)

where *d* is the input dimension. The probability formula for the attention factor of the *i*_*th*_ query is expressed as:

$$A(q_{i},K,V)=\sum_{j}\frac{k(q_{i},k_{i})}{\sum_{l}k(q_{i},k_{l})}v_{j}=E_{p}(k_{i}\left|q_{i}\right.)[V_{j}]$$ (18)

where *p(k*_*j*_*|q*_*i*_*)* is the probability distribution formula in the traditional transformer, *q(k*_*j*_*|q*_*i*_*)* is the uniform distribution, and *k(k*_*j*_*|q*_*i*_*)* is the asymmetric exponential kernel function.

In fact, the result after dot product obeys the long-tail distribution, which means that only the dot product result of a small number of queries and keys dominates, so other dot product results can be ignored, which can reduce computational complexity. Due to sparse self-attention matrix, some scholars calculated the relative entropy of the probability distribution of the attention mechanism of the query relative to the uniform distribution by using use the Kullback-Leibler(KL) scatter. The formula for evaluating the sparsity of the *i*_*th*_ query can be expressed as:

$$M(q_{i},K)=In\sum_{j=1}^{L_{K}}e^{\frac{q_{i}k_{j}{}^{T}}{\sqrt{d}}}-\frac{1}{L_{K}}\sum_{j=1}^{L_{K}}e^{\frac{q_{i}k_{j}{}^{T}}{\sqrt{d}}}$$ (19)

where the first term is the log-sum-exp of the inner product of *q*_*i*_ and all keys, and the second term is their arithmetic average. Thus when dot products U = L_Q_InL_K_, the complexity for computing $\bar{\text{M}}(q_{i},K)$ will be reduced from O(L_Q_L_K_) to L_Q_InL_K_, finally leading to such new ProbSpare self-attention expressed as:

$$Atten(Q,K,V)=Softmax(\frac{\bar{Q}K^{T}}{\sqrt{d}})V,Q\in R^{L_{Q}\times d},K\in R^{L_{K}\times d},V\in R^{L_{V}\times d}$$ (20)

where $\bar{Q}$ is a sparse matrix containing only the first *u* dominant queries, i.e., top *u* queries after selection. The sparse matrix of $\bar{Q}$ in the new and original attention mechanisms is of the same size.

*(2) Encoder*. Informer’s Encoder stack is a combination of multiple encoders and distillation layers. The purpose is to allow the encoder to process longer sequences of input by halving the individual layer features in the time dimension by means of an attentional distillation mechanism.

As a result of the ProbSpare self-attention, there are redundant combinations of feature mappings of encoder with value *V*. Therefore, distilling operation is performed to assign higher weights to dominant features with dominant attention and generate focus self-attention feature mappings at the next layer. The distilling operation process from *j* to *j+1* layers is expressed as:

$$X_{{}_{j+1}}^{t}=MaxPool(ELU(Convld({\left[X_{j}{}^{t}\right]}_{AB})))$$ (21)

**Fig 3:**
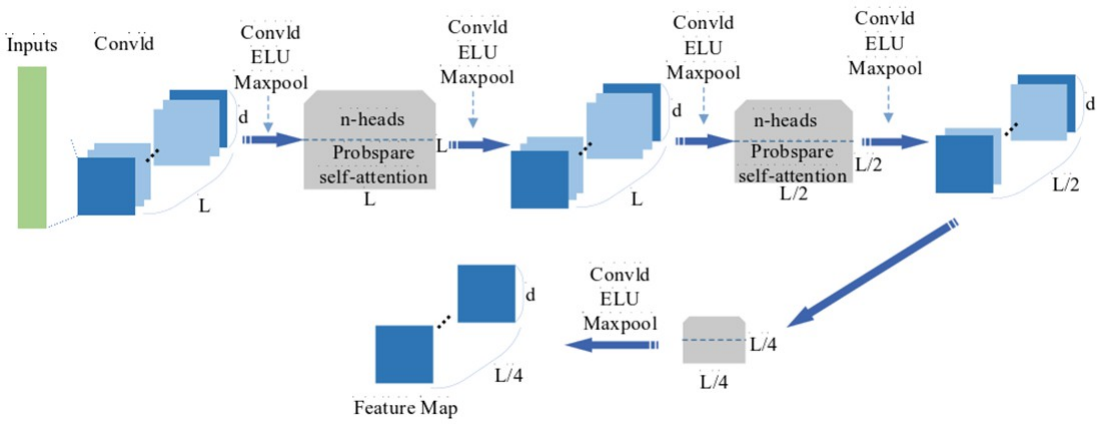
Calculation process of encoder.

This process involves multi-head probsparse self-attention and key operations in the attention block. Conv1d represents a one-dimensional convolutional operation on a time series with ELU as the activation function, followed by maximum pooling operation. To enhance the robustness of the attention distillation mechanism, the multiple halved copies of the main sequence are also constructed, with each being half the length of the previous one, which undergo the same attention distillation mechanism as the main sequence, constructing multiple feature maps of length L/4. Finally, these feature maps are stitched together into the final feature map of the input encoder. With the above method, the size of the feature maps can be gradually reduced without consuming too much memory in the computational space. The process of Encoder is briefed in Fig 3.

*(3) Decoder*. A decoder is added to the structure, comprising of two multi-headed self-attention layers. The probabilistic self-attention and canonical attention are respectively adopted for the first and second layers. The encoder output and the input sequence after embedding the projection serve as the input of the decoder. The input sequence is divided into two sections.
$$X_{{}_{feed\_dec}}^{t}=Concat(X_{{}_{token}}^{t},X_{{}_{phol}}^{t})\in R^{(L_{token}+L_{y})\times d_{model}},X_{{}_{token}}^{t}\in R^{L_{token}\times d_{model}}$$ (22)
  where *X*_*feed_dec*_ is the input sequence to the decoder; *X*_*token*_ is the start flag, and *X*_*phol*_ is the target placeholder.

Timestamps are padded with zeros to maintain dimensionality consistency during input in the prediction sequence. Masked multi-headed self-attention is applied to self-attention that masks future information. Each position focuses on current information and avoids auto-regression so that the model directly predicts all the outputs through a forward process without step-by-step dynamic decoding, resulting in a dramatic reduction in prediction decoding time.

### Long short-term memory (LSTM)

**Fig 4:**
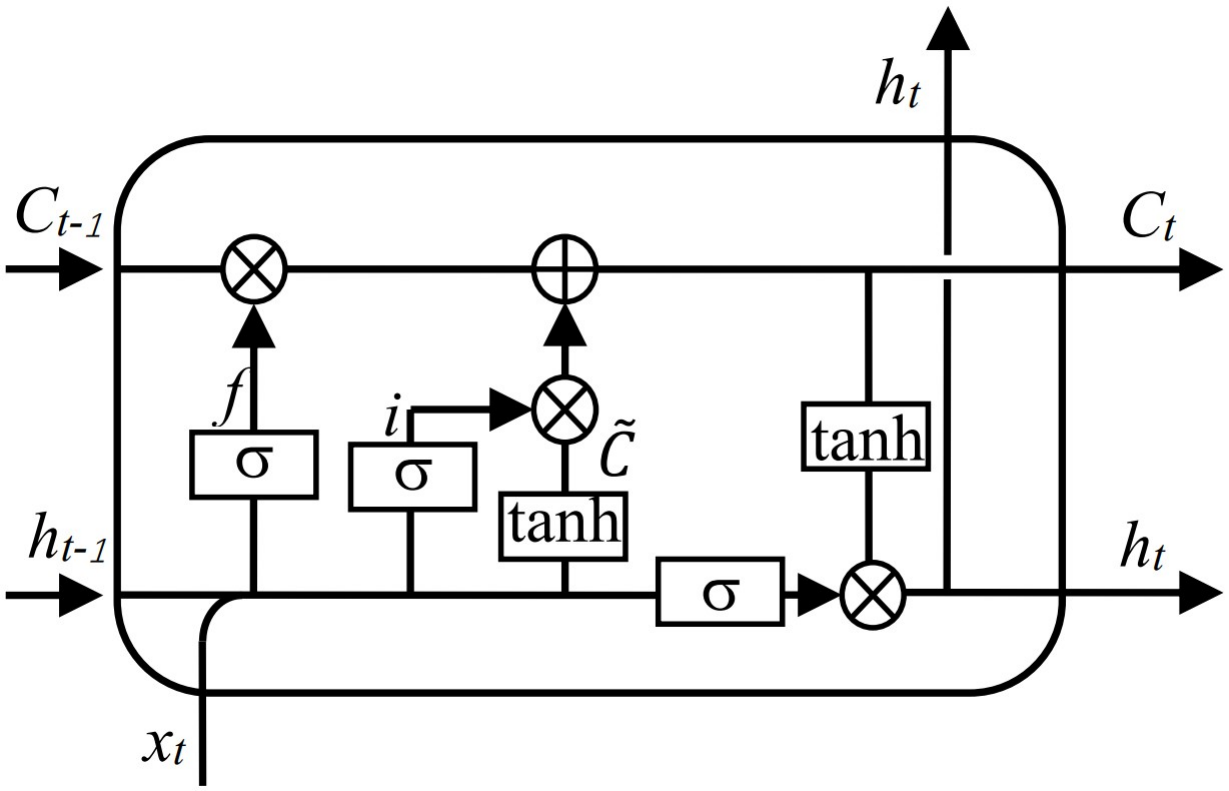
Structure diagram of LSTM.

Long Short-Term Memory (LSTM) is a deep learning model that has become popular in processing sequential data due to its ability to address the problem of long-term dependencies in traditional recurrent neural networks. LSTM introduces memory cells that contain both a hidden state and a memory state at each time step. The hidden state is used to pass information, while the memory state is used to store information. The gate mechanism is at the core of LSTM and comprises three gate units: input gate, forget gate, and output gate. These gates learn the state based on the current input and previous states and can be dynamically adjusted. The input gate controls which information should be added to the memory, the forget gate controls which information should be forgotten, and the output gate controls which information should be output from the memory. With this structure, LSTM can learn long-term dependencies in sequential data, and its application has been widespread in various fields, including speech recognition, natural language processing, and image processing. The structure of LSTM is shown in Fig 4.

#### Forgetting gate

The forgetting gate determines which information should be forgotten based on the sigmoid function. Its input includes the previous hidden state *h*_*t-1*_ and the current input *x*_*t*_, and the weight *W*_*f*_ is the weight of the previous layer of neurons. The forgetting gate is expressed as:

$$f_{t}=\sigma (W_{f}\cdot [h_{t}{}_{-1},x_{t}]+b_{f})$$ (23)

#### Input gate

The input gate determines which information should be retained and adds new memory. It is determined by a sigmoid layer and a subsequent tanh layer that generates a candidate value ($\tilde{C_{t}}$) to add to the neuronal state. The output gate *i*_*t*_ and the cell output $\tilde{C_{t}}$ are expressed in formulas:

$$i_{t}=\sigma (w_{i}\cdot [h_{t-1},x_{t}]+b_{i})$$ (24)

$$\tilde{C_{t}}=\text{tanh}(W_{C}\cdot [h_{t-1},x_{t}]+b_{C})$$ (25)

#### Output gate

The output gate determines the neuronal state *C*_*t*_ and how much information should be output in the input *o*_*t*_. The output gate *o*_*t*_ and the cell output *h*_*t*_ are expressed in formulas:

$$o_{t}=\sigma (W_{o}\cdot [h_{t-1},x_{t}]+b_{o})$$ (26)

$$h_{t}=o_{t}*\text{tanh}(C_{t})$$ (27)

In the above equations, *W*_*f*_, *W*_*i*_, *W*_*c*_, *W*_*o*_ and *b*_*f*_, *b*_*i*_, *b*_*c*_, *b*_*o*_ are the weights and biases of the forget gate, input gate, candidate value, and output gate, respectively.

#### Update neuron status

The top part of the LSTM structure updates the state of neurons from the previous state *C*_*t-1*_ to the new state *C*_*t*_ and then to *C*_*t+1*_. The update of its status is determined by the forgetting gate and input gate to decide which information to forget and retain. The formula for updating the state of neurons is expressed as:

$$C_{t}=f_{t}*C_{t-1}+i_{t}*\tilde{C_{t}}$$ (28)

### Analysis of combination mechanism

Informer adopts the network architecture of Transformer, which consists of multiple self-attention layers and feed-forward neural network layers. The self-attention mechanism employed by Informer allows the network to capture long-term dependencies within the sequence on a global scale, facilitating parallel computation and expediting model training and inference processes. In contrast, LSTM may encounter challenges such as gradient vanishing or exploding when dealing with lengthy sequential data, thereby hindering the effective capture of long-term dependencies. In comparison, the self-attention mechanism utilized by Informer enables a better capture of dependencies in long sequences while being less susceptible to the issues of gradient vanishing or exploding. LSTM’s recursive structure necessitates sequential computations at each time step, rendering parallelization unattainable. Conversely, the self-attention mechanism in the Informer network permits parallel computation across the entire sequence, thereby enhancing computational efficiency.

The decomposed sequence resulting from the decomposition algorithm exhibits characteristics of both high-frequency and low-frequency components. Considering the limitations of a single predictor in extracting features from the sequence, this study selects Informer, suitable for high-frequency components, and LSTM, suitable for low-frequency components, to design the Informer-LSTM combined prediction algorithm. To validate the effectiveness of the proposed combined algorithm, comparative experiments were conducted.

**Fig 5:**
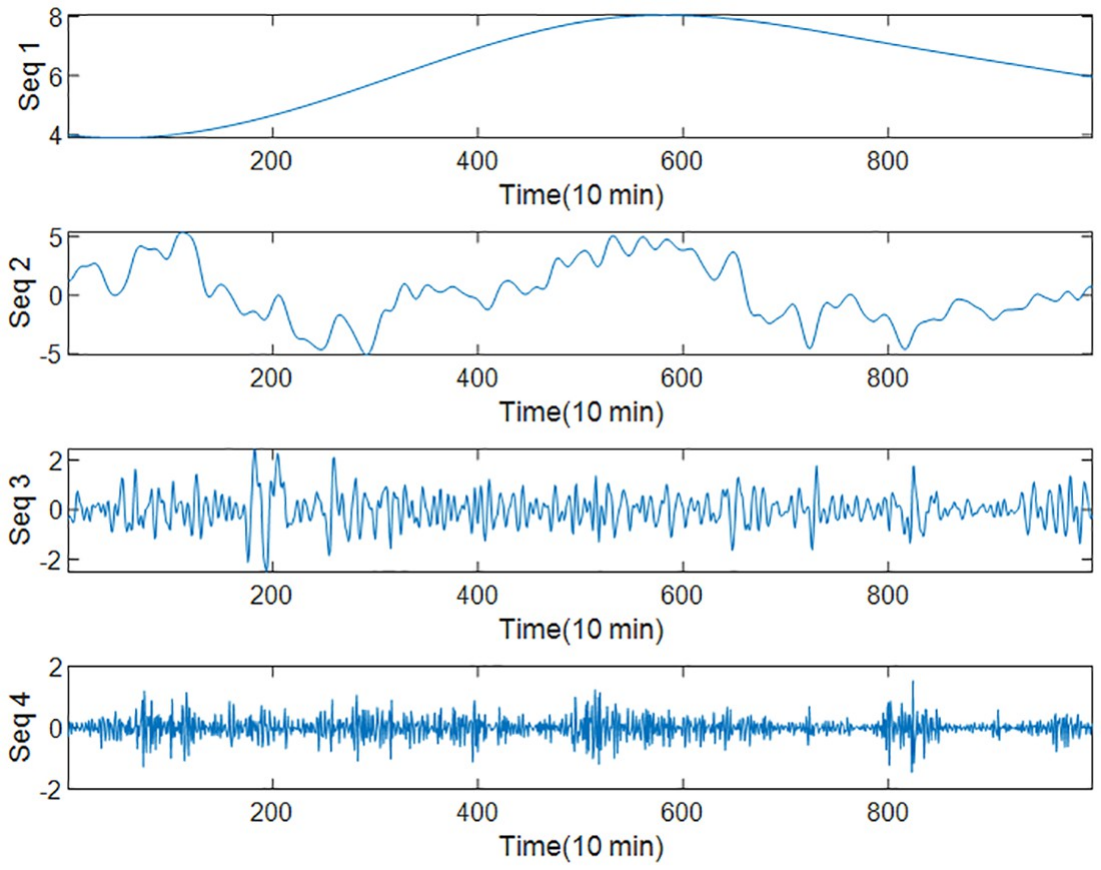
Decomposition of test wind speed set.

Based on part of the wind speed data set, a group of comparative experiments were set up, and LSTM and INFORMER were used to predict the four subsequences in Fig 5. The results are shown in Table 2.

**Table 2:** RMSE of each subsequence predicted by two predictors.

	Seq1	Seq2	Seq3	Seq4
**LSTM**	0.31967	**0.26136**	**0.16043**	**0.37599**
**INFORMER**	**0.13150**	0.38620	0.34800	0.71581

As shown in Table 2. LSTM is suitable for predicting low-frequency sequences with relatively small fluctuations, while INFORMER is more suitable for predicting high-frequency sequences with high volatility. Therefore, this article considers combining the advantages of both, predicting low-frequency sequences from LSTM and high-frequency sequences from INFORMER for the decomposed and recombined subsequences.

**Fig 6:**
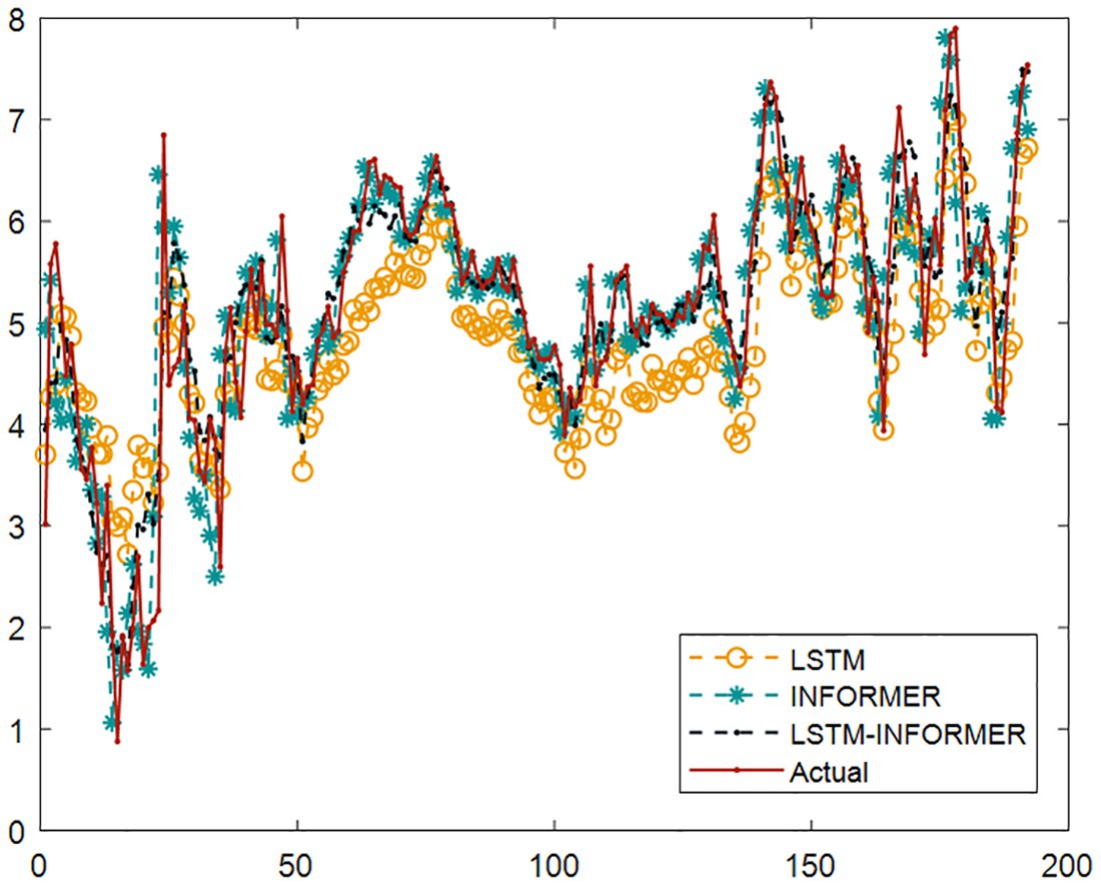
Comparison of test results.

To further validate its performance, a set of comparative experiments were conducted based on partial wind speed datasets, comparing LSTM INFORMER with individual LSTM and INFORMER. The predicted results are shown in Fig 6 and Table 3. It can be observed that LSTM INFORMER has higher prediction accuracy.

**Table 3:** Comparison of prediction accuracy of three models.

	LSTM	INFORMER	LSTM-INFORMER
RMSE	0.6204	0.4820	0.3143
MAE	0.7315	0.6973	0.4536

### Wind speed prediction model

**Fig 7:**
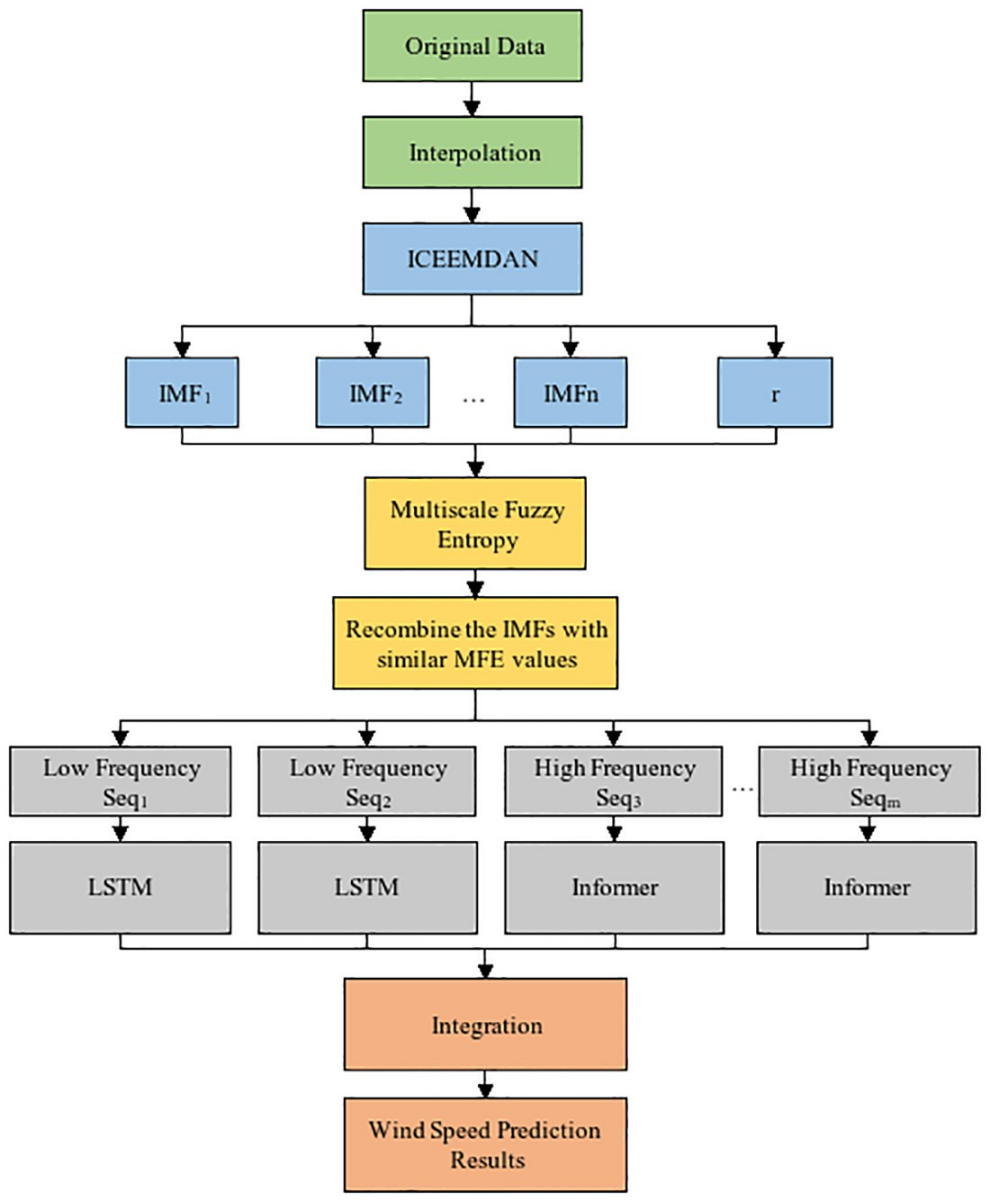
Framework of the proposed hybrid model.

Based on the principles and excellence of each algorithm introduced in Section 2, the ICEEMDAN-MFE-INFORMER hybrid prediction model is constructed. This section presents the whole framework and process of the hybrid prediction model to reduce the error in prediction results. The prediction process is shown in Fig 7.

Firstly, wind speed data are incomplete, and missing data are compensated by interpolation. Then complete continuous wind speed time series is decomposed into multiple intrinsic mode components as well as a residual by ICEEMDAN to make it less difficult to predict non-smooth series. The complexity of each component is evaluated by MFE, the MFE value of each IMF is calculated, and the IMF components with similar MFE values are reconstructed to obtain several new subseries. Finally, to predict each subsequence, LSTM and INFORMER are combined since they have different predictive effects on sequences with unknown complexity at different frequencies. After testing, it was observed that LSTM has better predictive performance for smooth sequences while INFORMER is more suitable for subsequences with larger fluctuations. Therefore, the predictor to be used is selected based on the degree of fluctuation of the subsequence. The prediction results of each subsequence are superimposed to obtain the final prediction results.

## Case study

In this section, the effectiveness of the proposed hybrid prediction model is demonstrated through a real case. All the experiments are implemented in python 3.9, pytorch 1.6.4, AMD Ryzen 7 6800H with Radeon Graphics, RAM 16G.

### Data source

In order to test the universality of the proposed model, this section selects two cases with different lengths and sampling frequencies for the simultaneous prediction of wind speed.

Case 1: The wind speed data in Case 1 were obtained from a wind farm with a 10-minute sampling interval over a 16-day period from March 14, 2022, to March 30, 2022. A total of 2448 sampling points were collected, and all missing data points were filled using interpolation.
Case 2: This dataset contains 2,390 wind speed data points, with no missing data. The data was recorded at an hourly sampling frequency, covering a period of 6 months from 20:00 on June 3, 2020 to 9:00 on September 11, 2020.

The statistical information of the wind speed datasets from two cases is listed in Table 4.

**Table 4:** The statistics of wind speed data sets in two cases.

Case	Samples	Groups	Statistical information(m/s)				
			Max	Min	Mean	Std	Median
Case1	All	2448	14.03	0.02	5.45	2.74	6.26
	Train set	1714	14.03	0.02	6.15	2.69	4.86
	Test set	734	10.04	0.02	3.80	2.08	5.21
Case2	All	2390	9.16	0.02	4.51	1.80	4.34
	Train set	1673	9.02	0.02	4.16	1.81	4.38
	Test set	717	9.16	0.03	5.35	1.46	5.37

Table 4 reveals noticeable volatility in the data for both cases. Specifically, Case 1 exhibits a wider range between the maximum and minimum values, accompanied by a larger standard deviation (STD). These observations indicate that Case 1 demonstrates higher volatility compared to Case 2, rendering prediction more challenging.

**Fig 8:**
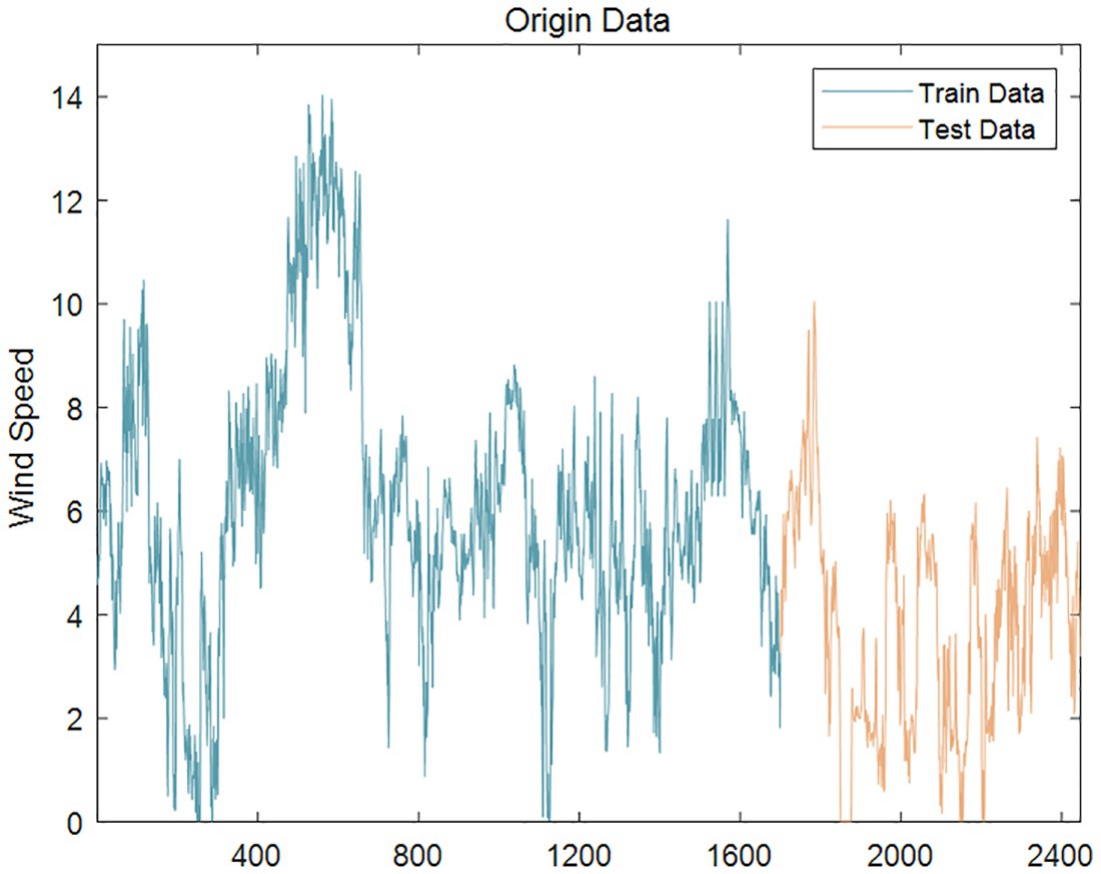
Wind speed series (Case 1).

**Fig 9:**
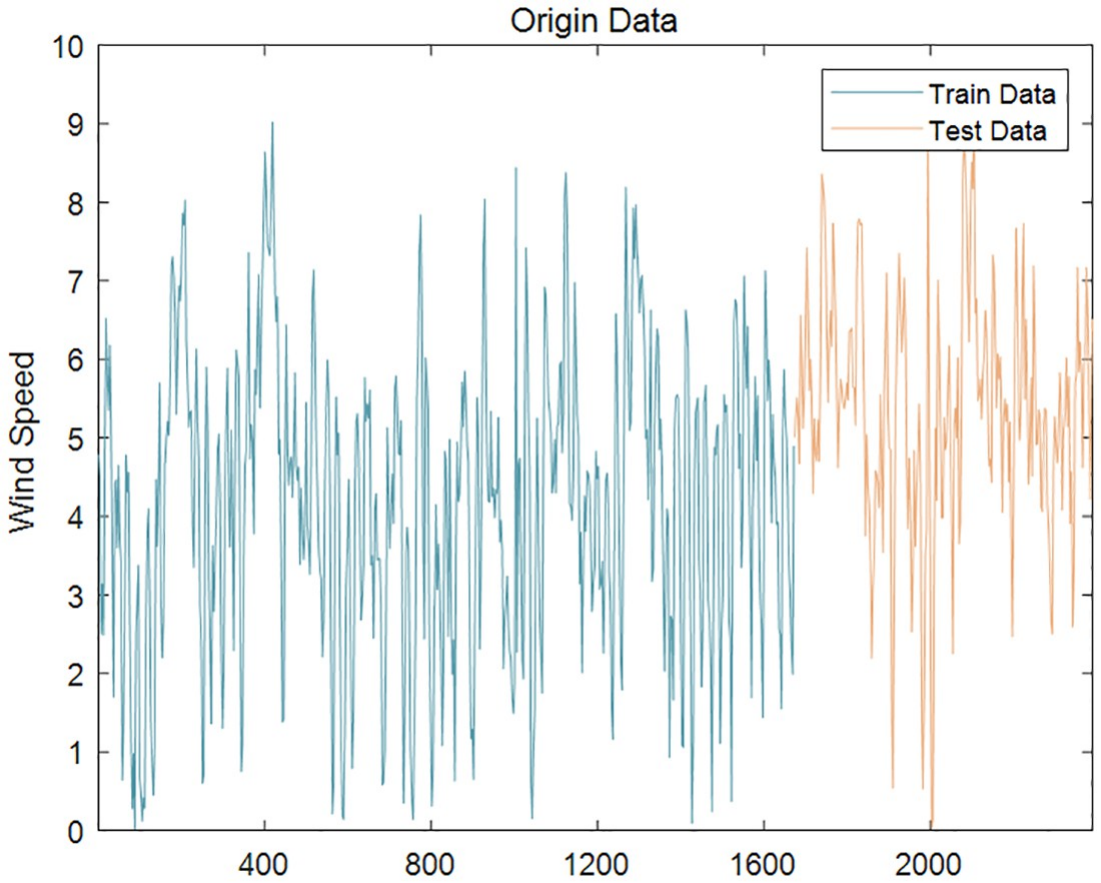
Wind speed series (Case2).

The unit root test (ADF) was performed on the wind speed series to analyze their volatility and non-stationarity. Due to the large sample size, direct ADF test would lead to excessive AIC value and poor fitting. Therefore, five sub-sequences with 100 decimal points were randomly selected from the original wind speed data to conduct ADF test respectively. The test results are shown in the Table 5. At the 0th order difference, the significance p-values of all five samples were greater than 0.05, indicating that the wind speed series were non-stationary. The first 70% of the data were used for training the model and the remaining 30% for testing. The original wind speed series are shown in Figs 8 and 9.

**Table 5:** P-value of ADF test for each sequence.

	Subseqs	Seq1	Seq2	Seq3	Seq4	Seq5
Case1	p-value	0.676	0.512	0.701	0.667	0.584
Case2	p-value	0.710	0.779	0.643	0.801	0.757

### Evaluation index

To verify the superiority of the model, four evaluation metrics, namely root mean square error (RMSE), mean absolute error (MAE), mean absolute percentage error (MAPE), and coefficient of determination R2 were selected and calculated by:

$$RMSE(\tilde{y_{n}},y_{n})=\sqrt{\frac{1}{N}\cdot {\sum_{n=1}^{N}\left(y_{n}-\tilde{y_{n}}\right)}^{2}}$$ (29)

$$MAE(\tilde{y_{n}},y_{n})=\frac{1}{N}\cdot \sum_{n=1}^{N}\left|y_{n}-\tilde{y_{n}}\right|$$ (30)

$$MAPE(\tilde{y_{n}},y_{n})=\frac{1}{N}\sum_{n=1}^{N}\left|\frac{y_{n}-\tilde{y_{n}}}{y_{n}}\right|$$ (31)

$$R^{2}(\tilde{y_{n}},y_{n})=1-\frac{\sum_{n=1}^{N}{\left(y_{n}-\tilde{y_{n}}\right)}^{2}}{\sum_{n=1}^{N}{\left(y_{n}-\bar{y}\right)}^{2}}$$ (32)

where *y*_*n*_ is the actual value, ${\tilde{y}}_{n}$ is the predicted value, ${\bar{y}}_{n}$ is the average value, and N is the number of predicted sequence points.

### Parameter setting and comparison model

The wind speed sequence was predicted by the informer structure, with the above mentioned four(five in case 2) wind speed subsequences as the input and the prediction result of wind speed as the output. The parameters of the informer structure were set as follows in Table 6.

**Table 6:** Main parameter settings for each model.

Methods	Parameter	Value	Methods	Parameter	Value
INFORMER	Time feature encoding frequency	10t (i.e., 10min)(1hour in case2)	LSTM	maximum training number	1800
	model dimension	32		learning rate	0.005
	number of heads of multi-head self-attention	8		number of hidden layers	1
	number of encoder layers	2	RF	delay step	15
	number of decoder layers	1		number of decision trees	100
	number of stacked encoder layers	3		minimum number of leaves	5
	number of sampling factors	5	ICEEMDAN	The standard deviation	0.2
	attention mechanism of encoding	prob		the maximum screening number	5000
	dropout	0.05	CEEMDAN	white noise standard deviation	0.2
	experimental times	5		maximum number of filters	5000
	initial learning rate	0.001	VMD	bandwidth limit	2000
	input sequence length	96		noise tolerance	0.3
	start token length	48		modes	9(11 in case2)
	prediction sequence length	24	MFE	Embedding Dimension	2
	loss function	mean square error		Time Delay	1
				the similarity tolerance	0.2

The proposed model based on ICEEMDAN-MFE-LSTM-INFORMER was compared with seven relevant models, namely, ICEEMDAN-MFE-INFORMER, ICEEMDAN-MFE-LSTM, ICEEMDAN-MFE-RF, VMD-MFE-LSTM, CEEMDAN-MFE-LSTM, INFORMER, LSTM and RF, to verify its effectiveness in predicting wind speed. The parameters of these comparison models are set as follow in Table 6.

### Decomposition and recombination

**Fig 10:**
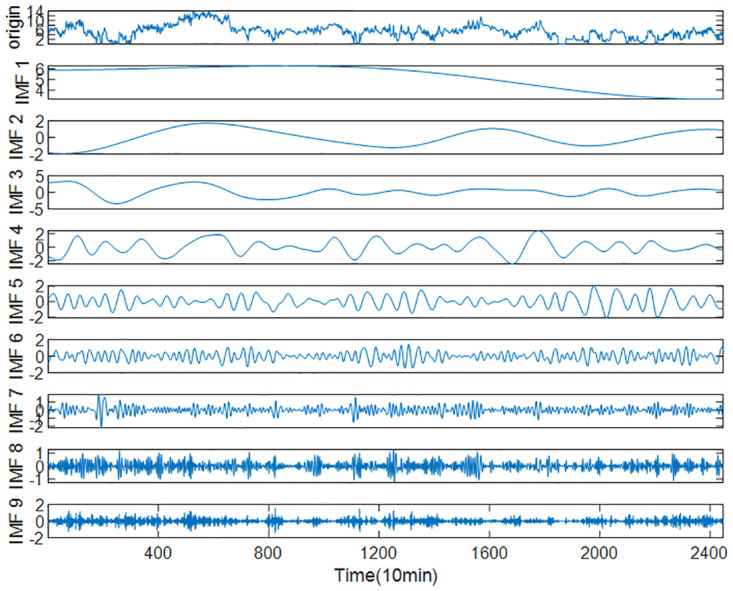
Decomposition results of wind speed series by ICEEMDAN (Case 1).

**Fig 11:**
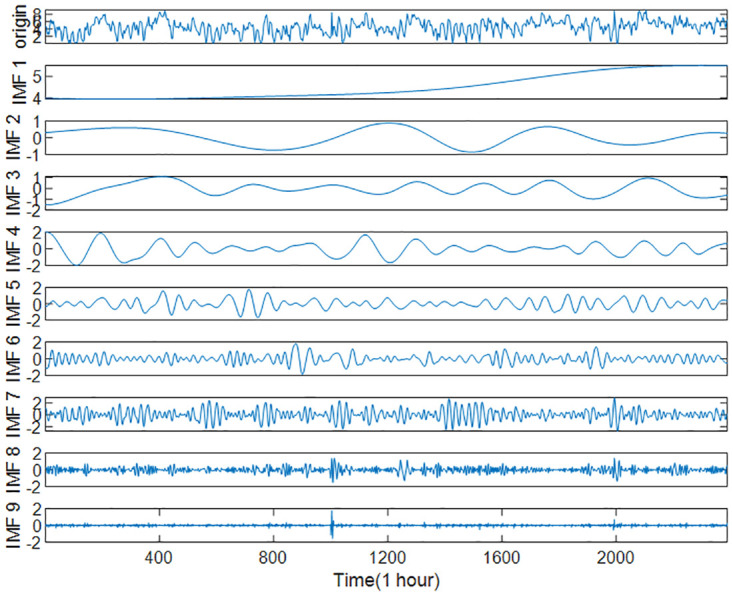
Decomposition results of wind speed series by ICEEMDAN (Case2).

The complete wind speed series was obtained by interpolation method and decomposed by ICEEMDAN method. The standard deviation *Nstd* was set to 0.2 and the maximum screening number *Maxlter* to 5000 according to several experiments and reasonable optimization. The decomposition results are shown in Figs 10 and 11, where the first term is the initial wind speed data, and the remaining nine(eleven in case 2) terms are decomposed IMFs (the last item is a residual r) arranged from high frequency to low frequency.

**Fig 12:**
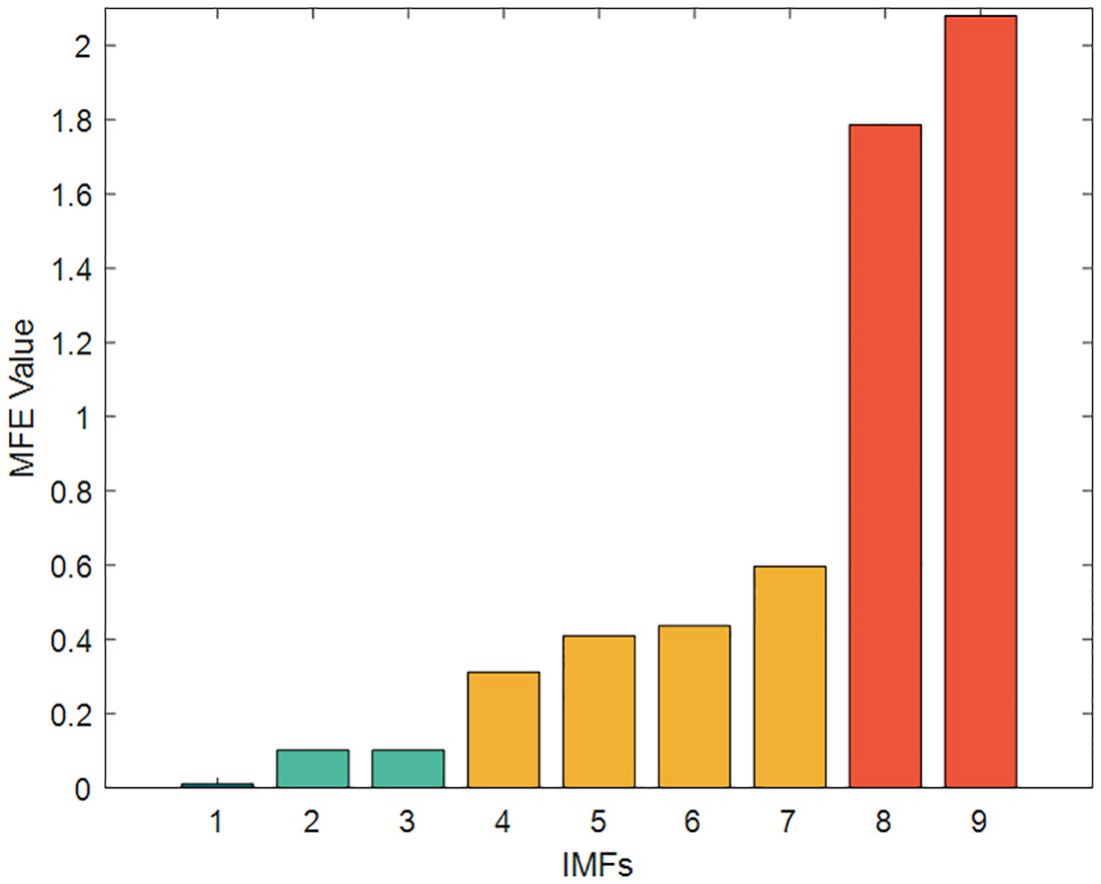
The MFE value of IMFs (Case1).

**Fig 13:**
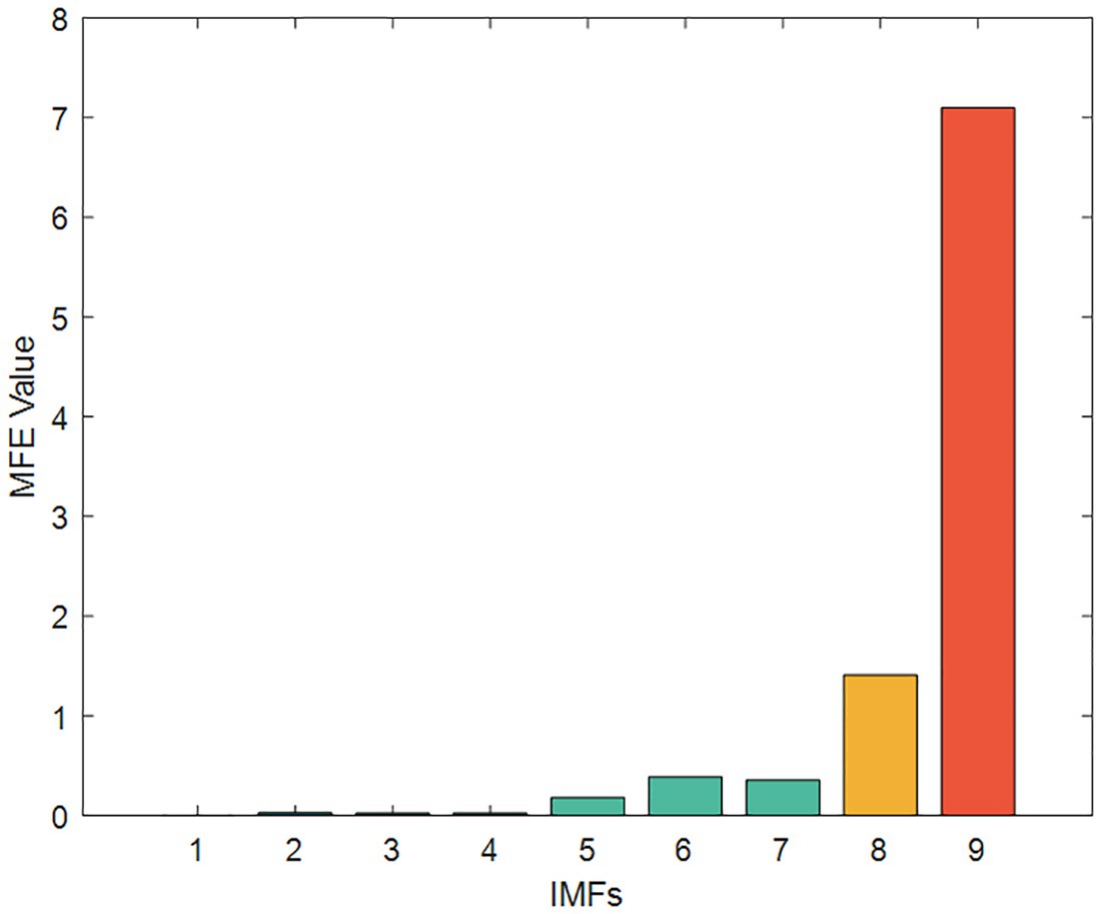
The MFE value of IMFs (Case2).

**Fig 14:**
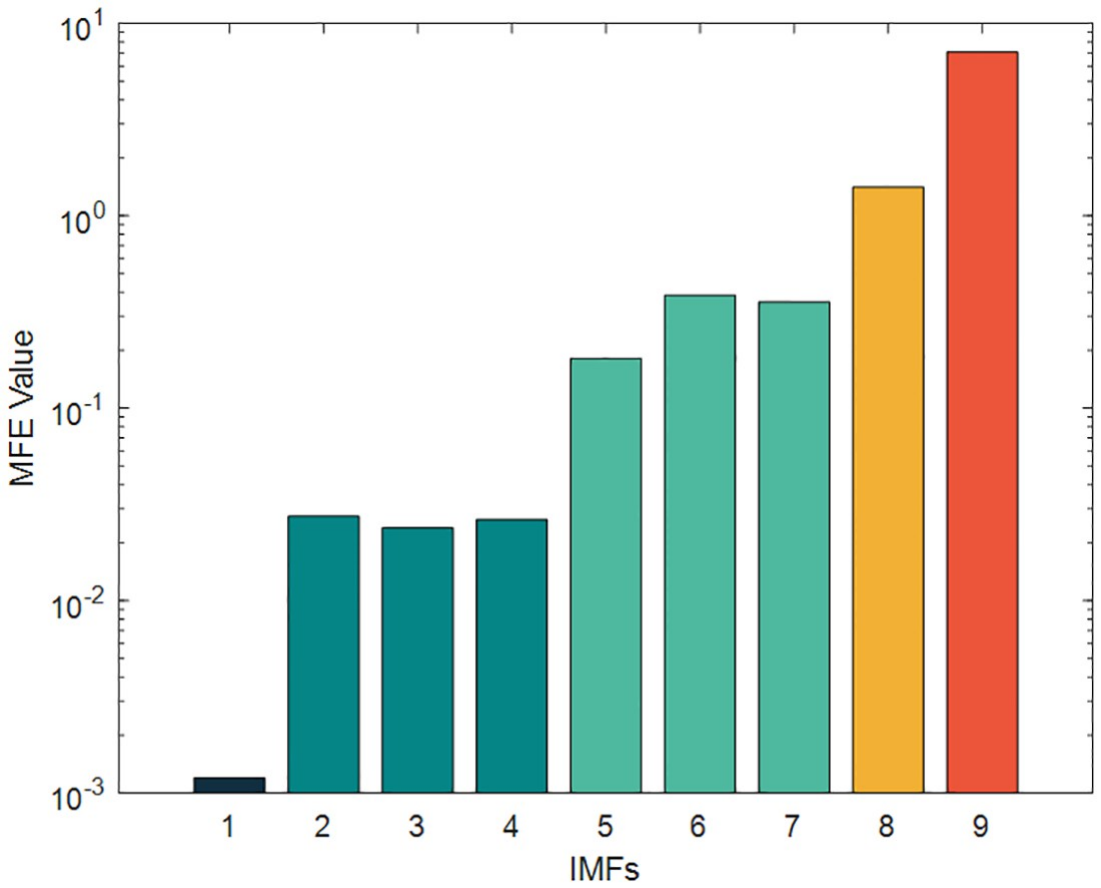
The MFE value of IMFs (Index chart of Case2).

**Fig 15:**
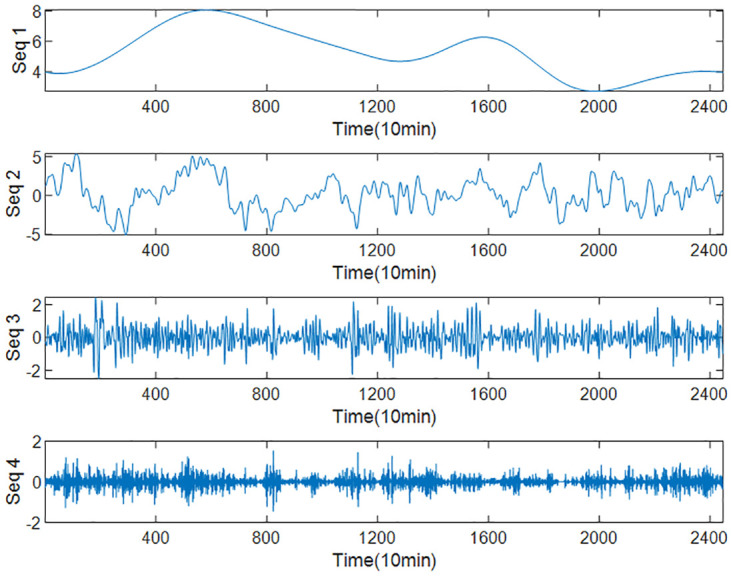
New sequence diagram of IMFs recombination (Case 1).

**Fig 16:**
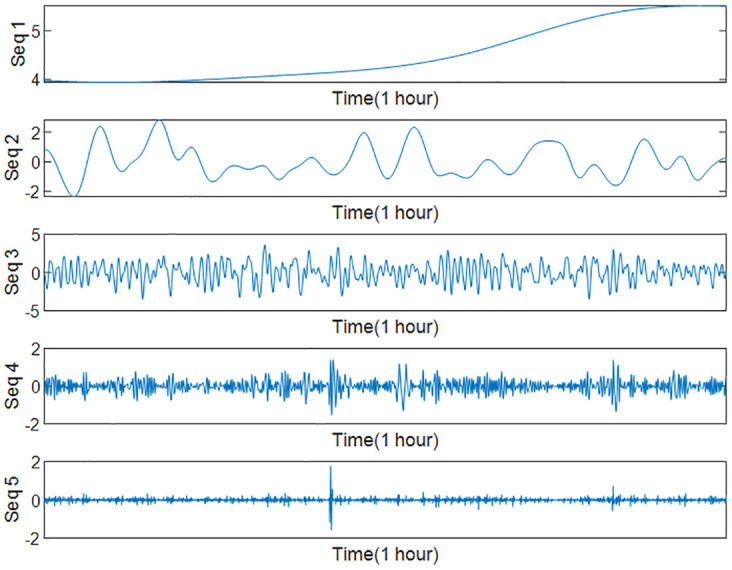
New sequence diagram of IMFs recombination (Case2).

To reduce the computational effort and time, the above nine IMFs were reasonably reconstructed by multi-scale fuzzy entropy under the premise of ensuring the prediction accuracy. The embedding dimension *m* was set to 2, the time_delay to 1, and the similarity tolerance *r* to 0.2 Std. The MFE values of each IMF were calculated, as shown in Table 7 and Figs 12–14 respectively. The IMFs with similar MFE values were reorganized and superimposed, as also respectively indicated in the table and figure. After being decomposed and recombined by ICEEMDAN and MFE methods, four(five in case 2) new subsequences were derived, as displayed in Table 8 and Figs 15 and 16.

**Table 7:** MFE values of IMFs.

Case1		Case2	
IMFs	MFE Value	IMFs	MFE Value
IMF1	0.01047	IMF1	0.0012
IMF2	0.10193	IMF2	0.0274
IMF3	0.10151	IMF3	0.0239
IMF4	0.31154	IMF4	0.0264
IMF5	0.41028	IMF5	0.1808
IMF6	0.43657	IMF6	0.3868
IMF7	0.59657	IMF7	0.3561
IMF8	1.78678	IMF8	1.4093
IMF9	2.07965	IMF9	7.0981

**Table 8:** The recombined sequence.

Case1	Seqs	Seq1	Seq2	Seq3	Seq4	**\**
	Combination	IMF1	IMF2,3	IMF4-7	IMF8,9	\
Case2	Seqs	Seq1	Seq2	Seq3	Seq4	Seq5
	Combination	IMF1	IMF2,3,4	IMF5,6,7	IMF8	IMF9

### Analysis of prediction results

Based on the decomposition and reconstruction method described above, each subsequence is predicted by a combination of LSTM and INFORMER. LSTM is used for smoother sequences, while INFORMER is used for sequences with larger fluctuations. The predictor selected for each subsequence is presented in Table 9. The final wind speed prediction result is obtained by combining the prediction results of each subsequence.

**Table 9:** The effect of two kinds of predictors on different subsequences.

Case1	Seqs	Seq1	Seq2	Seq3	Seq4
	Predictor	LSTM	INFORMER	INFORMER	INFORMER
Case2	Seqs	Seq1	Seq2	Seq3	Seq4
	Predictor	LSTM	LSTM	INFORMER	INFORMER

**Fig 17:**
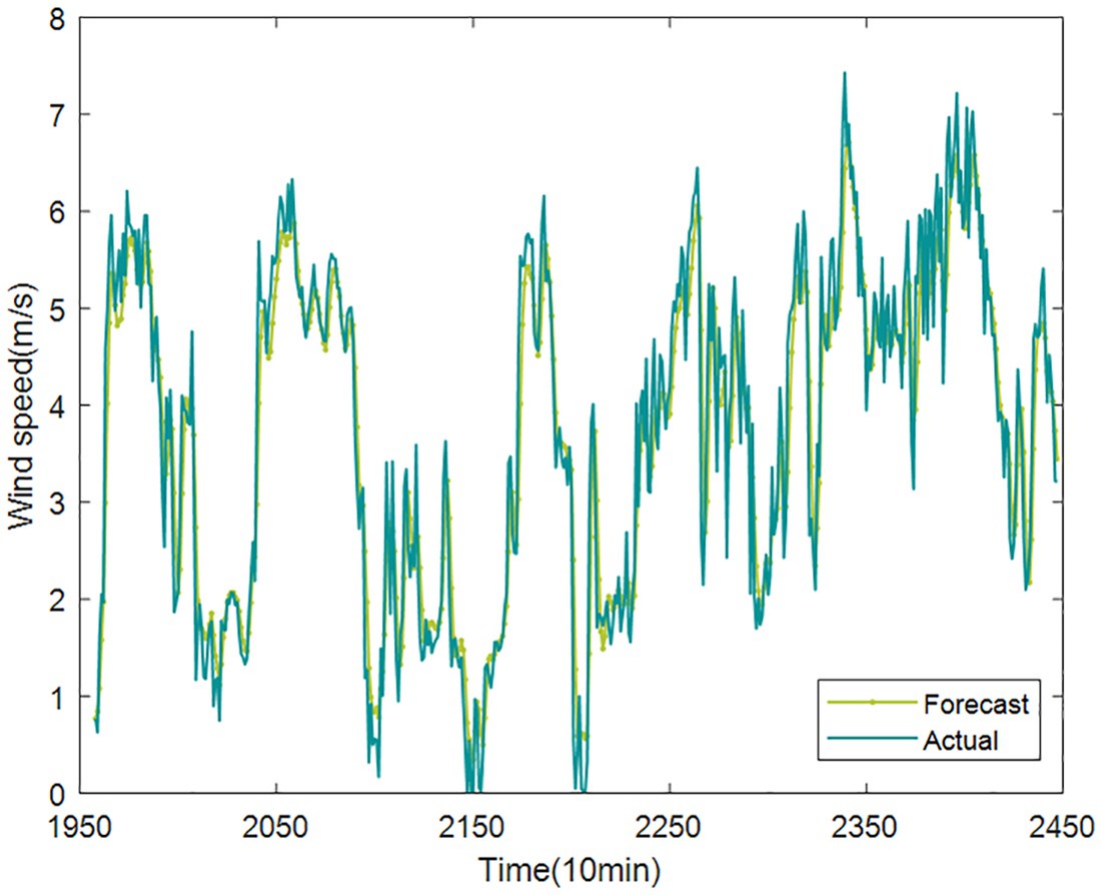
The final prediction results of wind speed (Case1).

**Fig 18:**
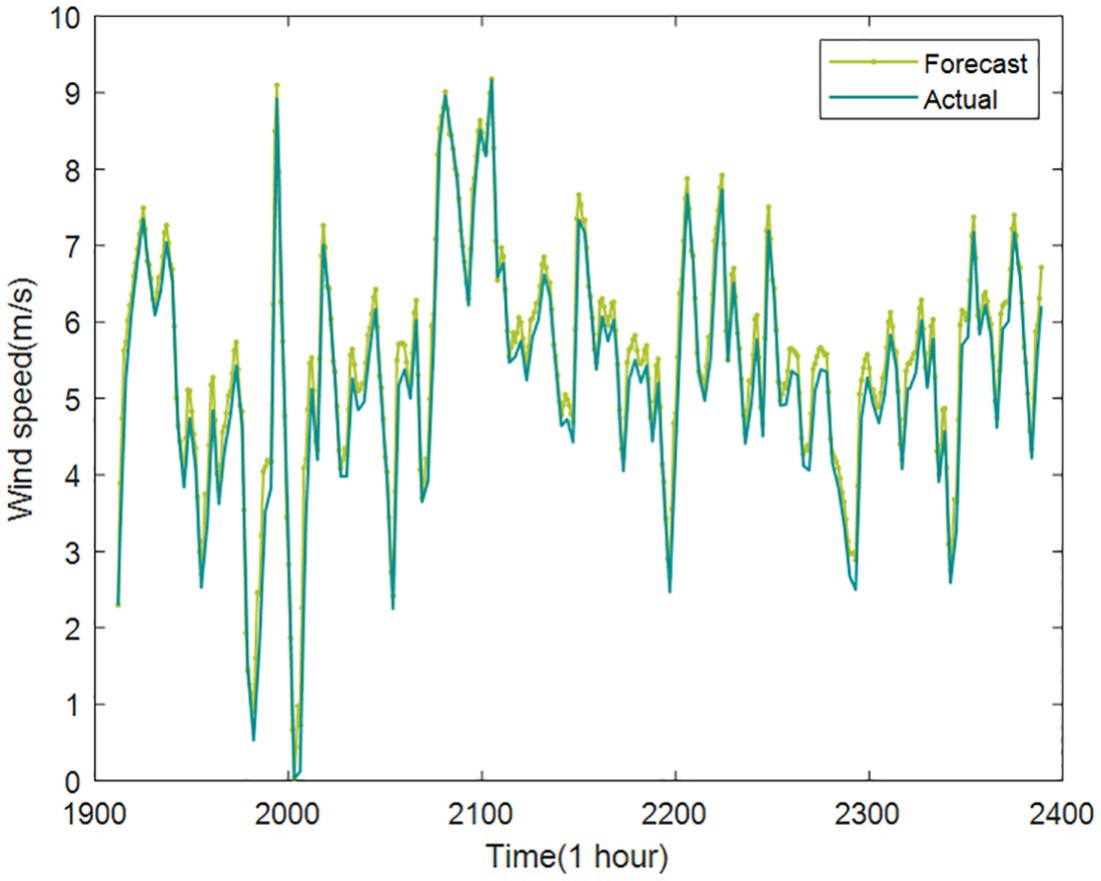
The final prediction results of wind speed (Case2).

Based on the above decomposition and reconstruction, the final prediction results of wind speed are shown in Figs 17 and 18.

To evaluate the efficacy of the proposed hybrid model, a series of comparative experiments were conducted. Specifically, the following comparisons were made: (1) comparison of a single predictive model, (2) Comparison of decomposition mechanisms, (3) Comparison of mixed prediction models. These comparisons were conducted to analyze the performance of the hybrid model in relation to other methods.

The four evaluation indexes (RMSE, MAE, MAPE, and R2) of each model were calculated separately. Their prediction accuracy is indicated below.

#### (1) Comparison of a single predictive model

Comparison of single prediction models including RF, LSTM, and INFORMER, and the comparison results are shown in Table 10.

**Table 10:** Comparison of a single predictive model.

	Models	RMSE	MAE	MAPE	R^2^
Case1	RF	1.2443	0.9058	0.3574	0.6843
	LSTM	0.6503	0.5542	0.1604	0.8540
	INFORMER	0.6417	0.5334	0.1524	0.8643
Case2	RF	0.7264	0.5580	0.1957	0.8443
	LSTM	0.6931	0.5536	0.2164	0.8623
	INFORMER	0.6682	0.5317	0.2090	0.8713

#### (2) Comparison of decomposition mechanisms

The comparison of decomposition mechanisms including VMD-MFE-LSTM, CEEMDAN-MFE-LSTM, and ICEEMDAN-MFE-LSTM, and the comparison results are shown in Table 11.

**Table 11:** Comparison of decomposition mechanisms.

	Models	RMSE	MAE	MAPE	R^2^
Case1	VMD-MFE-LSTM	0.5590	0.3961	0.1467	0.8852
	CEEMDAN-MFE-LSTM	0.4936	0.4094	0.1268	0.8806
	ICEEMDAN-MFE-LSTM	0.4881	0.3937	0.1203	0.8895
Case2	VMD-MFE-LSTM	0.4394	0.3315	0.1467	0.9392
	CEEMDAN-MFE-LSTM	0.3531	0.2474	0.1207	0.9622
	ICEEMDAN-MFE-LSTM	0.3834	0.3290	0.1288	0.9595

#### (3) Comparison of mixed prediction models

The comparison of mixed prediction models including ICEEMDAN-MFE-RF, ICEEMDAN-MFE-LSTM, ICEEMDAN-MFE-INFORMER, ICEEMDAN-MFE-LSTM-INFORMER, and the comparison results are shown in Table 12.

**Table 12:** Comparison of mixed prediction models.

	Models	RMSE	MAE	MAPE	R^2^
Case1	ICEEMDAN-MFE-RF	0.6117	0.5063	0.1372	0.8797
	ICEEMDAN-MFE-LSTM	0.4881	0.3937	0.1203	0.8895
	ICEEMDAN-MFE-INFORMER	0.4936	0.4094	0.1268	0.8806
	ICEEMDAN-MFE-LSTM-INFORMER	0.4573	0.3878	0.1291	0.9411
Case2	ICEEMDAN-MFE-RF	0.5037	0.4091	0.1302	0.8846
	ICEEMDAN-MFE-LSTM	0.3834	0.3290	0.1288	0.9595
	ICEEMDAN-MFE-INFORMER	0.3531	0.2474	0.1207	0.9622
	ICEEMDAN-MFE-LSTM-INFORMER	0.3171	0.2142	0.1149	0.9653

Based on the above observations and calculation results, we drew the following conclusions.

1. The ICEEMDAN-MFE-LSTM-INFORMER prediction model exhibited higher accuracy with less error than the other seven models and better performance in predicting wind speed.
2. The hybrid prediction model using modal decomposition algorithm was more effective in predicting wind speed than single model as wind speed is generally non-stationary and difficult to directly predict. However, the modal composition makes it less difficult to predict wind speed as it filters out noise series in the wind speed. The above results indicated that the prediction accuracy of wind speed could be effectively improved by preprocessing data based on data decomposition.
3. ICEEMDAN outperformed VMD in terms of data decomposition due to its ability to address the issue of residual noise and pseudo-modality in the modal decomposition. Therefore, it was able to reduce the interference of noise sequences and improve the prediction accuracy.
4. The LSTM model exhibits higher prediction accuracy for smoother and less volatile sequences, whereas the INFORMER model is better suited for sequences with higher volatility.

## Conclusion

This study presents a hybrid prediction model that integrates ICEEMDAN, MFE, LSTM, and INFORMER to enhance the accuracy and reliability of wind speed prediction. Initially, the wind speed series is decomposed into intrinsic mode functions (IMF) using the ICEEMDAN decomposition algorithm, which separates the data into IMFs ranging from high to low frequencies. Subsequently, the MFE values of each IMF are computed, and IMFs with similar MFE values are reconstructed, yielding multiple new subsequences. Next, the combination mechanism of LSTM and INFORMER is analyzed. LSTM is found to be suitable for modeling relatively stable low-frequency sequences, whereas INFORMER performs better in capturing high-frequency sequences with significant fluctuations. Accordingly, the combined model is employed to predict the decomposed and reconstructed subsequences. LSTM is employed for smoother series, while INFORMER is utilized for more volatile series. Ultimately, the predicted values of each subsequence are combined to generate the final prediction results. To assess the effectiveness of the proposed prediction model, a comparative analysis is conducted with seven alternative prediction models. The experimental results, evaluated using multiple indicators, confirm the superior performance and efficacy of the proposed combination method.

Precise wind speed prediction facilitates enhanced management of renewable energy resources, optimized energy market transactions, improved power system scheduling and operation, as well as effective planning and risk management of wind energy projects. Accurate wind speed prediction further enables improved energy efficiency, reduced energy costs, and decreased reliance on conventional energy sources. Consequently, the proposed hybrid model holds substantial theoretical and practical significance.

Nonetheless, several issues remain to be addressed in future research. Currently, few scholars have considered influential factors on wind speed, such as seasonal variations, atmospheric conditions, meteorological systems, and measurement heights. Therefore, incorporating these factors into wind speed prediction poses a major challenge. It is imperative to select appropriate prediction methods based on different scenarios and make adjustments based on actual conditions, thereby enhancing the overall credibility and reliability of the predictions.

## Abbreviations

ADGWDTO: Adaptive Dynamic Grey Wolf-Dipper Throated Optimization
ARIMA: Autoregressive integrated moving average
ARMA: Auto regression moving average
Bi-LSTM: Bidirectional LSTM
CNN: Convolutional Neural Network
ConvLSTM: Convolutional LSTM
EEMD: Ensemble EMD
ELM: Extreme Learning Machine
EMD: Empirical Mode Decomposition
FE: Fuzzy Entropy
FWNSDEC: filter-wrapper non-dominated sorting differential evolution integrating K-medoid clustering
GARCH: generalized autoregressive conditional heteroskedasticity
HTD: hybrid time series decomposition
ICEEMDAN: Improved Complete Ensemble Empirical Mode Decomposition with Adaptive Noise
IMFs: Intrinsic Mode Functions
LSTM: Long short-term memory
MFE: Multiscale Fuzzy Entropy
MKSVRE: multi-kernel SVR ensemble
MOBBSA: multi-objective binary backtracking search algorithm
MSE: Mean Squared Error
NWP: Numerical Weather Prediction
OVMD: Optimal VMD
RNN: Recurrent Neural Network
RWT-ARIMA: Repeated Wavelet Transform with ARIMA
SE: Sample Entropy
SVM: Support Vector Machine
SVR: Support vector regression
VMD: Variational mode decomposition
WOA: whale optimization algorithm
